# 2-((*Z*)-{3-[(*Z*)-(2-Hy­droxy-5-methyl­benzyl­idene)amino]-2,2-dimethyl­prop­yl}imino­meth­yl)-4-methyl­phenol

**DOI:** 10.1107/S1600536810051688

**Published:** 2010-12-15

**Authors:** Reza Kia, Hadi Kargar, Valiollah Mirkhani, Fatemeh Ganji, Muhammad Nawaz Tahir

**Affiliations:** a Department of Chemistry, Science and Research Branch, Islamic Azad University, Tehran, Iran; b X-ray Crystallography Laboratory, Plasma Physics Research Center, Science and Research Branch, Islamic Azad University, Tehran, Iran; c Department of Chemistry, School of Science, Payame Noor University (PNU), Ardakan, Yazd, Iran; d Department of Chemistry, University of Isfahan, Isfahan, 81746-73441, Iran; e Department of Physics, University of Sargodha, Punjab, Pakistan

## Abstract

In the title compound, C_21_H_26_N_2_O_2_, the dihedral angle between the two benzene rings is 73.47 (16)°. Strong intra­molecular O—H⋯N hydrogen bonds generate *S*(6) ring motifs. The substituted benzene rings are twisted around the central quaternary C atom in opposite directions, making a vault geometry.

## Related literature

For standard bond lengths, see: Allen *et al.* (1987 ▶). For hydrogen-bond motifs, see: Bernstein *et al.* (1995 ▶). For related structures, see: Kargar *et al.* (2009 ▶, 2010 ▶).
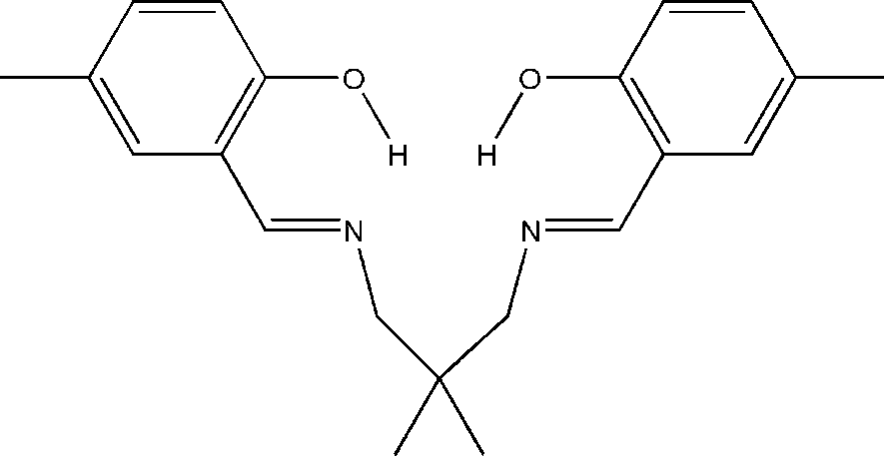


## Experimental

### 

#### Crystal data

C_21_H_26_N_2_O_2_
*M* *_r_* = 338.44
Orthorhombic, 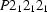

*a* = 5.8950 (3) Å
*b* = 17.8634 (10) Å
*c* = 18.2140 (11) Å
*V* = 1918.02 (19) Å^3^
*Z* = 4
Mo *K*α radiation
μ = 0.08 mm^−1^
*T* = 296 K
0.30 × 0.18 × 0.12 mm

#### Data collection

Bruker SMART APEXII CCD area-detector diffractometer
Absorption correction: multi-scan (*SADABS*; Bruker, 2005 ▶) *T*_min_ = 0.978, *T*_max_ = 0.991
16208 measured reflections
2199 independent reflections
1368 reflections with *I* > 2σ(*I*)
*R*_int_ = 0.062

#### Refinement

*R*[*F* ^2^ > 2σ(*F* ^2^)] = 0.044
*wR*(*F* ^2^) = 0.112
*S* = 1.03
2199 reflections
231 parameters
H-atom parameters constrained
Δρ_max_ = 0.15 e Å^−3^
Δρ_min_ = −0.12 e Å^−3^

### 

Data collection: *APEX2* (Bruker, 2005 ▶); cell refinement: *SAINT* (Bruker, 2005 ▶); data reduction: *SAINT*; program(s) used to solve structure: *SHELXTL* (Sheldrick, 2008 ▶); program(s) used to refine structure: *SHELXTL*; molecular graphics: *SHELXTL*; software used to prepare material for publication: *SHELXTL* and *PLATON* (Spek, 2009 ▶).

## Supplementary Material

Crystal structure: contains datablocks global, I. DOI: 10.1107/S1600536810051688/gw2096sup1.cif

Structure factors: contains datablocks I. DOI: 10.1107/S1600536810051688/gw2096Isup2.hkl

Additional supplementary materials:  crystallographic information; 3D view; checkCIF report

## Figures and Tables

**Table 1:** Hydrogen-bond geometry (Å, °)

*D*—H⋯*A*	*D*—H	H⋯*A*	*D*⋯*A*	*D*—H⋯*A*
O1—H1*A*⋯N1	0.82	1.89	2.620 (3)	147
O2—H2*A*⋯N2	0.82	1.88	2.609 (4)	147

**Fig. 1.**
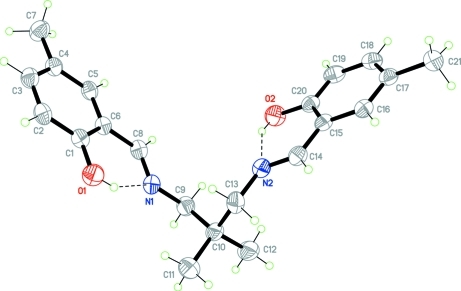
The asymmetric unit of the title compound, showing 30% probability displacement ellipsoids and the atomic numbering. Intramolecular hydrogen bonds are drawn as dashed lines.

## supplementary crystallographic information

### Comment

Schiff base ligands are one of the most prevalent systems in coordination chemistry. As part of a general study of tetradenate Schiff bases (Kargar *et al.* 2009; Kargar *et al.* 2010), we have determined the crystal structure of the title compound.

The asymmetric unit of the title compound, Fig. 1, comprises a potentially tetradenate Schiff base ligand. The bond lengths (Allen *et al.,* 1987) and angles are within the normal ranges. The dihedral angle between the two phenyl rings is 73.47 (16)°. Strong intramolecular O—H···N hydrogen bonds generate *S(6)* ring motifs (Bernstein *et al.,* 1995). The title compound has a skew geometry. In the absence of sufficient anomalous scattering the absolute structure could not be determined.

### Experimental

The title compound was synthesized by adding 5-methyl-salicylaldehyde (4 mmol) to a solution of 2,2'-dimethylpropylenediamine (2 mmol) in ethanol (20 ml). The mixture was refluxed with stirring for half an hour. The resultant yellow solution was filtered. Yellow single crystals of the title compound suitable for *X*-ray structure determination were recrystallized from ethanol by slow evaporation of the solvents at room temperature over several days.

### Refinement

H atoms of the hydroxy groups were located by a rotating O–H group and constraied to refine with the parent atoms with U_iso_(H) = 1.5 U_eq_(O), see Table 1. The remaining H atoms were positioned geometrically with C—H = 0.93-0.97 Å and included in a riding model approximation with U_iso_ (H) = 1.2 or 1.5 U_eq_ (C). A rotating group model was used for the methyl groups of the benzene rings. In the absence of sufficient anomalous scattering the absolute structure could not be determined and 1580 Friedel pairs were merged.

### Crystal data

**Table:**

C_21_H_26_N_2_O_2_	*F*(000) = 728
*M**_r_* = 338.44	*D*_x_ = 1.172 Mg m^−^^3^
Orthorhombic, *P*2_1_2_1_2_1_	Mo *K*α radiation, λ = 0.71073 Å
Hall symbol: P 2ac 2ab	Cell parameters from 2150 reflections
*a* = 5.8950 (3) Å	θ = 2.5–29.8°
*b* = 17.8634 (10) Å	µ = 0.08 mm^−^^1^
*c* = 18.2140 (11) Å	*T* = 296 K
*V* = 1918.02 (19) Å^3^	Plate, yellow
*Z* = 4	0.30 × 0.18 × 0.12 mm

### Data collection

**Table:**

Bruker SMART APEXII CCD area-detector diffractometer	2199 independent reflections
Radiation source: fine-focus sealed tube	1368 reflections with *I* > 2σ(*I*)
graphite	*R*_int_ = 0.062
φ and ω scans	θ_max_ = 26.0°, θ_min_ = 2.2°
Absorption correction: multi-scan (*SADABS*; Bruker, 2005)	*h* = −7→7
*T*_min_ = 0.978, *T*_max_ = 0.991	*k* = −22→22
16208 measured reflections	*l* = −22→22

### Refinement

**Table:**

Refinement on *F*^2^	Secondary atom site location: difference Fourier map
Least-squares matrix: full	Hydrogen site location: inferred from neighbouring sites
*R*[*F*^2^ > 2σ(*F*^2^)] = 0.044	H-atom parameters constrained
*wR*(*F*^2^) = 0.112	*w* = 1/[σ^2^(*F*_o_^2^) + (0.0485*P*)^2^ + 0.082*P*] where *P* = (*F*_o_^2^ + 2*F*_c_^2^)/3
*S* = 1.03	(Δ/σ)_max_ < 0.001
2199 reflections	Δρ_max_ = 0.15 e Å^−^^3^
231 parameters	Δρ_min_ = −0.12 e Å^−^^3^
0 restraints	Extinction correction: *SHELXTL* (Sheldrick, 2008), Fc^*^=kFc[1+0.001xFc^2^λ^3^/sin(2θ)]^-1/4^
Primary atom site location: structure-invariant direct methods	Extinction coefficient: 0.013 (2)

### Special details

**Table:**

Geometry. All esds (except the esd in the dihedral angle between two l.s. planes) are estimated using the full covariance matrix. The cell esds are taken into account individually in the estimation of esds in distances, angles and torsion angles; correlations between esds in cell parameters are only used when they are defined by crystal symmetry. An approximate (isotropic) treatment of cell esds is used for estimating esds involving l.s. planes.
Refinement. Refinement of F^2^ against ALL reflections. The weighted R-factor wR and goodness of fit S are based on F^2^, conventional R-factors R are based on F, with F set to zero for negative F^2^. The threshold expression of F^2^ > 2sigma(F^2^) is used only for calculating R-factors(gt) etc. and is not relevant to the choice of reflections for refinement. R-factors based on F^2^ are statistically about twice as large as those based on F, and R- factors based on ALL data will be even larger.

### Fractional atomic coordinates and isotropic or equivalent isotropic displacement parameters (Å^2^)

**Table:**

	*x*	*y*	*z*	*U*_iso_*/*U*_eq_	
O1	0.4633 (4)	0.71477 (13)	0.18637 (14)	0.0691 (7)	
H1A	0.3887	0.6786	0.1999	0.104*	
O2	−0.1798 (4)	0.44857 (14)	0.02145 (14)	0.0769 (7)	
H2A	−0.0936	0.4643	0.0533	0.115*	
N1	0.1130 (4)	0.62471 (14)	0.19160 (15)	0.0576 (7)	
N2	0.1758 (5)	0.44863 (14)	0.10671 (15)	0.0589 (7)	
C1	0.3362 (5)	0.75772 (18)	0.14103 (17)	0.0513 (8)	
C2	0.4215 (5)	0.82499 (18)	0.11644 (19)	0.0593 (9)	
H2	0.5650	0.8401	0.1316	0.071*	
C3	0.2994 (6)	0.87012 (19)	0.07010 (18)	0.0593 (9)	
H3	0.3614	0.9153	0.0545	0.071*	
C4	0.0828 (5)	0.84926 (18)	0.04596 (18)	0.0558 (9)	
C5	−0.0004 (6)	0.78143 (17)	0.07017 (17)	0.0544 (8)	
H5	−0.1421	0.7659	0.0537	0.065*	
C6	0.1183 (5)	0.73540 (17)	0.11802 (17)	0.0487 (8)	
C7	−0.0504 (7)	0.90009 (19)	−0.0039 (2)	0.0777 (11)	
H7A	0.0048	0.8952	−0.0533	0.117*	
H7B	−0.0332	0.9510	0.0120	0.117*	
H7C	−0.2079	0.8865	−0.0023	0.117*	
C8	0.0162 (6)	0.66623 (17)	0.14440 (18)	0.0562 (9)	
H8	−0.1246	0.6520	0.1261	0.067*	
C9	−0.0035 (6)	0.55714 (17)	0.21706 (19)	0.0641 (10)	
H9A	−0.1067	0.5401	0.1791	0.077*	
H9B	−0.0929	0.5693	0.2601	0.077*	
C10	0.1605 (6)	0.49409 (17)	0.23599 (18)	0.0615 (9)	
C11	0.3201 (7)	0.5184 (2)	0.2980 (2)	0.0892 (13)	
H11A	0.2336	0.5276	0.3417	0.134*	
H11B	0.3982	0.5633	0.2838	0.134*	
H11C	0.4286	0.4794	0.3072	0.134*	
C12	0.0218 (8)	0.4259 (2)	0.2593 (2)	0.0891 (13)	
H12A	0.1216	0.3842	0.2673	0.134*	
H12B	−0.0850	0.4136	0.2214	0.134*	
H12C	−0.0582	0.4370	0.3039	0.134*	
C13	0.3085 (6)	0.47518 (18)	0.16952 (19)	0.0640 (10)	
H13A	0.3929	0.5194	0.1551	0.077*	
H13B	0.4171	0.4369	0.1833	0.077*	
C14	0.2562 (6)	0.39671 (18)	0.06619 (18)	0.0571 (9)	
H14	0.3962	0.3762	0.0784	0.069*	
C15	0.1397 (5)	0.36831 (16)	0.00220 (18)	0.0512 (8)	
C16	0.2388 (6)	0.31298 (17)	−0.04090 (17)	0.0542 (8)	
H16	0.3790	0.2940	−0.0267	0.065*	
C17	0.1400 (6)	0.28490 (17)	−0.10341 (18)	0.0570 (9)	
C18	−0.0714 (6)	0.3128 (2)	−0.1220 (2)	0.0638 (10)	
H18	−0.1438	0.2943	−0.1636	0.077*	
C19	−0.1782 (6)	0.3673 (2)	−0.08042 (19)	0.0648 (9)	
H19	−0.3205	0.3850	−0.0941	0.078*	
C20	−0.0727 (5)	0.39531 (18)	−0.01880 (19)	0.0542 (9)	
C21	0.2588 (7)	0.22644 (19)	−0.14975 (19)	0.0775 (12)	
H21A	0.3779	0.2497	−0.1775	0.116*	
H21B	0.1517	0.2039	−0.1827	0.116*	
H21C	0.3222	0.1886	−0.1184	0.116*	

### Atomic displacement parameters (Å^2^)

**Table:**

	*U*^11^	*U*^22^	*U*^33^	*U*^12^	*U*^13^	*U*^23^
O1	0.0584 (15)	0.0711 (17)	0.0778 (17)	0.0012 (12)	−0.0145 (14)	0.0031 (14)
O2	0.0690 (16)	0.0818 (17)	0.0798 (19)	0.0272 (15)	0.0009 (14)	−0.0003 (14)
N1	0.0590 (17)	0.0501 (16)	0.0636 (18)	0.0000 (13)	0.0014 (15)	−0.0032 (14)
N2	0.0615 (17)	0.0523 (17)	0.0629 (18)	0.0032 (14)	−0.0008 (16)	0.0023 (15)
C1	0.0439 (18)	0.0545 (19)	0.056 (2)	0.0055 (16)	−0.0042 (16)	−0.0062 (16)
C2	0.0503 (19)	0.060 (2)	0.068 (2)	−0.0066 (17)	−0.0024 (18)	−0.0138 (18)
C3	0.057 (2)	0.056 (2)	0.065 (2)	−0.0064 (17)	0.0018 (18)	−0.0047 (18)
C4	0.056 (2)	0.054 (2)	0.058 (2)	0.0018 (16)	0.0007 (17)	−0.0054 (16)
C5	0.0467 (18)	0.055 (2)	0.061 (2)	−0.0020 (16)	−0.0037 (17)	−0.0096 (17)
C6	0.0423 (17)	0.0497 (18)	0.0540 (19)	−0.0008 (14)	0.0011 (16)	−0.0062 (16)
C7	0.078 (3)	0.070 (2)	0.085 (3)	−0.0059 (19)	−0.015 (2)	0.014 (2)
C8	0.0485 (19)	0.057 (2)	0.063 (2)	−0.0020 (16)	0.0052 (17)	−0.0098 (17)
C9	0.067 (2)	0.061 (2)	0.064 (2)	−0.0107 (19)	0.0100 (19)	−0.0015 (17)
C10	0.072 (2)	0.052 (2)	0.060 (2)	−0.0139 (19)	−0.0039 (19)	0.0081 (17)
C11	0.109 (3)	0.080 (3)	0.079 (3)	−0.012 (2)	−0.027 (3)	−0.002 (2)
C12	0.116 (3)	0.077 (3)	0.074 (3)	−0.032 (3)	−0.009 (3)	0.016 (2)
C13	0.060 (2)	0.052 (2)	0.080 (3)	−0.0010 (17)	−0.006 (2)	−0.0061 (18)
C14	0.0495 (19)	0.054 (2)	0.068 (2)	0.0020 (16)	−0.0012 (18)	0.0062 (18)
C15	0.0506 (19)	0.0491 (18)	0.0538 (19)	0.0021 (15)	0.0049 (17)	0.0105 (16)
C16	0.0496 (19)	0.0534 (19)	0.059 (2)	0.0028 (15)	0.0031 (17)	0.0083 (17)
C17	0.063 (2)	0.055 (2)	0.053 (2)	−0.0015 (17)	0.0069 (18)	0.0096 (17)
C18	0.064 (2)	0.072 (2)	0.055 (2)	−0.0100 (19)	−0.0029 (19)	0.0109 (18)
C19	0.0495 (19)	0.079 (3)	0.066 (2)	0.0022 (19)	−0.002 (2)	0.018 (2)
C20	0.051 (2)	0.0555 (19)	0.057 (2)	0.0067 (16)	0.0082 (17)	0.0106 (17)
C21	0.099 (3)	0.068 (2)	0.065 (2)	0.011 (2)	0.006 (2)	−0.0031 (19)

### Geometric parameters (Å, °)

**Table:**

O1—C1	1.354 (3)	C10—C12	1.527 (5)
O1—H1A	0.8200	C10—C13	1.530 (4)
O2—C20	1.357 (4)	C10—C11	1.533 (5)
O2—H2A	0.8200	C11—H11A	0.9600
N1—C8	1.271 (4)	C11—H11B	0.9600
N1—C9	1.464 (4)	C11—H11C	0.9600
N2—C14	1.276 (4)	C12—H12A	0.9600
N2—C13	1.465 (4)	C12—H12B	0.9600
C1—C2	1.377 (4)	C12—H12C	0.9600
C1—C6	1.409 (4)	C13—H13A	0.9700
C2—C3	1.371 (4)	C13—H13B	0.9700
C2—H2	0.9300	C14—C15	1.445 (4)
C3—C4	1.401 (4)	C14—H14	0.9300
C3—H3	0.9300	C15—C16	1.391 (4)
C4—C5	1.380 (4)	C15—C20	1.395 (4)
C4—C7	1.506 (4)	C16—C17	1.374 (4)
C5—C6	1.388 (4)	C16—H16	0.9300
C5—H5	0.9300	C17—C18	1.384 (5)
C6—C8	1.456 (4)	C17—C21	1.515 (4)
C7—H7A	0.9600	C18—C19	1.386 (5)
C7—H7B	0.9600	C18—H18	0.9300
C7—H7C	0.9600	C19—C20	1.377 (4)
C8—H8	0.9300	C19—H19	0.9300
C9—C10	1.524 (5)	C21—H21A	0.9600
C9—H9A	0.9700	C21—H21B	0.9600
C9—H9B	0.9700	C21—H21C	0.9600
			
C1—O1—H1A	109.5	C10—C11—H11B	109.5
C20—O2—H2A	109.5	H11A—C11—H11B	109.5
C8—N1—C9	119.0 (3)	C10—C11—H11C	109.5
C14—N2—C13	119.3 (3)	H11A—C11—H11C	109.5
O1—C1—C2	119.4 (3)	H11B—C11—H11C	109.5
O1—C1—C6	121.7 (3)	C10—C12—H12A	109.5
C2—C1—C6	118.9 (3)	C10—C12—H12B	109.5
C3—C2—C1	121.4 (3)	H12A—C12—H12B	109.5
C3—C2—H2	119.3	C10—C12—H12C	109.5
C1—C2—H2	119.3	H12A—C12—H12C	109.5
C2—C3—C4	121.0 (3)	H12B—C12—H12C	109.5
C2—C3—H3	119.5	N2—C13—C10	112.6 (3)
C4—C3—H3	119.5	N2—C13—H13A	109.1
C5—C4—C3	117.2 (3)	C10—C13—H13A	109.1
C5—C4—C7	122.5 (3)	N2—C13—H13B	109.1
C3—C4—C7	120.3 (3)	C10—C13—H13B	109.1
C4—C5—C6	122.8 (3)	H13A—C13—H13B	107.8
C4—C5—H5	118.6	N2—C14—C15	123.0 (3)
C6—C5—H5	118.6	N2—C14—H14	118.5
C5—C6—C1	118.6 (3)	C15—C14—H14	118.5
C5—C6—C8	120.1 (3)	C16—C15—C20	117.9 (3)
C1—C6—C8	121.3 (3)	C16—C15—C14	120.4 (3)
C4—C7—H7A	109.5	C20—C15—C14	121.7 (3)
C4—C7—H7B	109.5	C17—C16—C15	123.3 (3)
H7A—C7—H7B	109.5	C17—C16—H16	118.3
C4—C7—H7C	109.5	C15—C16—H16	118.3
H7A—C7—H7C	109.5	C16—C17—C18	117.0 (3)
H7B—C7—H7C	109.5	C16—C17—C21	121.2 (3)
N1—C8—C6	122.2 (3)	C18—C17—C21	121.9 (3)
N1—C8—H8	118.9	C17—C18—C19	121.9 (4)
C6—C8—H8	118.9	C17—C18—H18	119.0
N1—C9—C10	112.6 (3)	C19—C18—H18	119.0
N1—C9—H9A	109.1	C20—C19—C18	119.7 (3)
C10—C9—H9A	109.1	C20—C19—H19	120.2
N1—C9—H9B	109.1	C18—C19—H19	120.2
C10—C9—H9B	109.1	O2—C20—C19	119.0 (3)
H9A—C9—H9B	107.8	O2—C20—C15	120.8 (3)
C9—C10—C12	108.2 (3)	C19—C20—C15	120.2 (3)
C9—C10—C13	110.2 (3)	C17—C21—H21A	109.5
C12—C10—C13	110.4 (3)	C17—C21—H21B	109.5
C9—C10—C11	110.3 (3)	H21A—C21—H21B	109.5
C12—C10—C11	110.5 (3)	C17—C21—H21C	109.5
C13—C10—C11	107.2 (3)	H21A—C21—H21C	109.5
C10—C11—H11A	109.5	H21B—C21—H21C	109.5
			
O1—C1—C2—C3	179.6 (3)	C14—N2—C13—C10	−142.8 (3)
C6—C1—C2—C3	−0.3 (5)	C9—C10—C13—N2	−61.8 (4)
C1—C2—C3—C4	−0.1 (5)	C12—C10—C13—N2	57.8 (4)
C2—C3—C4—C5	−0.6 (5)	C11—C10—C13—N2	178.1 (3)
C2—C3—C4—C7	179.0 (3)	C13—N2—C14—C15	−178.5 (3)
C3—C4—C5—C6	1.7 (5)	N2—C14—C15—C16	178.3 (3)
C7—C4—C5—C6	−177.9 (3)	N2—C14—C15—C20	−1.2 (5)
C4—C5—C6—C1	−2.1 (4)	C20—C15—C16—C17	1.3 (4)
C4—C5—C6—C8	176.9 (3)	C14—C15—C16—C17	−178.3 (3)
O1—C1—C6—C5	−178.6 (3)	C15—C16—C17—C18	−1.7 (5)
C2—C1—C6—C5	1.3 (4)	C15—C16—C17—C21	177.9 (3)
O1—C1—C6—C8	2.4 (4)	C16—C17—C18—C19	1.0 (5)
C2—C1—C6—C8	−177.7 (3)	C21—C17—C18—C19	−178.7 (3)
C9—N1—C8—C6	178.2 (3)	C17—C18—C19—C20	0.2 (5)
C5—C6—C8—N1	−175.7 (3)	C18—C19—C20—O2	−179.8 (3)
C1—C6—C8—N1	3.3 (5)	C18—C19—C20—C15	−0.7 (5)
C8—N1—C9—C10	147.8 (3)	C16—C15—C20—O2	179.1 (3)
N1—C9—C10—C12	−178.1 (3)	C14—C15—C20—O2	−1.3 (5)
N1—C9—C10—C13	−57.2 (4)	C16—C15—C20—C19	0.0 (4)
N1—C9—C10—C11	60.9 (4)	C14—C15—C20—C19	179.5 (3)

### Hydrogen-bond geometry (Å, °)

**Table:**

*D*—H···*A*	*D*—H	H···*A*	*D*···*A*	*D*—H···*A*
O1—H1A···N1	0.82	1.89	2.620 (3)	147
O2—H2A···N2	0.82	1.88	2.609 (4)	147
